# Downregulation of the vitamin D receptor expression during acute gastrointestinal graft versus host disease is associated with poor outcome after allogeneic stem cell transplantation

**DOI:** 10.3389/fimmu.2022.1028850

**Published:** 2022-10-20

**Authors:** Carina Matos, Andreas Mamilos, Pranali N. Shah, Elisabeth Meedt, Daniela Weber, Saroj Ghimire, Andreas Hiergeist, André Gessner, Anne Dickinson, Ralf Dressel, Lutz Walter, Klaus Stark, Iris M. Heid, Hendrik Poeck, Matthias Edinger, Daniel Wolff, Wolfgang Herr, Ernst Holler, Marina Kreutz, Sakhila Ghimire

**Affiliations:** ^1^ Department of Internal Medicine III, University Hospital Regensburg, Regensburg, Germany; ^2^ Department of Pathology, University of Regensburg, Regensburg, Germany; ^3^ Department of Dermatology, Brigham and Women’s Hospital, Harvard Medical School, Boston, MA, United States; ^4^ Institute of Cellular and Molecular Immunology, University Medical Centre Göttingen, Göttingen, Germany; ^5^ Kathmandu University School of Medical Sciences, Dhulikhel, Nepal; ^6^ Institute of Clinical Microbiology and Hygiene, University Hospital Regensburg, Regensburg, Germany; ^7^ Translational and Clinical Research Institute, Faculty of Medical Sciences, Newcastle University, Newcastle upon Tyne, United Kingdom; ^8^ Primate Genetics Laboratory, German Primate Center, Leibniz-Institute for Primate Research, Göttingen, Germany; ^9^ Department for Genetic Epidemiology, University of Regensburg, Regensburg, Germany; ^10^ Leibniz Institute for Immunotherapy (LIT), Regensburg, Germany

**Keywords:** VDR, HSCT, acute GI-GvHD, TRM, defensins

## Abstract

The vitamin D receptor (VDR) is critical in regulating intestinal homeostasis and emerging evidence demonstrates that VDR deficiency is a critical factor in inflammatory bowel disease pathology. However, no clinical data exist regarding the intestinal expression of VDR in patients after allogeneic haematopoietic stem cell transplantation (HSCT). Analyzing intestinal biopsies from 90 patients undergoing HSCT with mortality follow-up, we demonstrated that patients with severe acute gastrointestinal graft versus host disease (GI-GvHD) showed significant downregulation of VDR gene expression compared to mild or no acute GI-GvHD patients (p = 0.007). Reduced VDR expression was already detectable at acute GI-GvHD onset compared to GvHD-free patients (p = 0.01). These results were confirmed by immunohistochemistry (IHC) where patients with severe acute GI-GvHD showed fewer VDR+ cells (p = 0.03) and a reduced VDR staining score (p = 0.02) as compared to mild or no acute GI-GvHD patients. Accordingly, low VDR gene expression was associated with a higher cumulative incidence of treatment-related mortality (TRM) (p = 1.6x10-6) but not with relapse-related mortality (RRM). A multivariate Cox regression analysis identified low VDR as an independent risk factor for TRM (p = 0.001, hazard ratio 4.14, 95% CI 1.78-9.63). Furthermore, VDR gene expression significantly correlated with anti-microbial peptides (AMPs) gene expression (DEFA5: r = 0.637, p = 7x10-5, DEFA6: r 0 0.546, p = 0.001). In conclusion, our findings suggest an essential role of the VDR in the pathogenesis of gut GvHD and the prognosis of patients undergoing HSCT.

## Graphical Abstract

Vitamin D receptor (VDR), a ligand-activated nuclear hormone receptor, is predominantly expressed in epithelial cells. During homeostasis, sufficient expression of VDR leads to a strong epithelial barrier and increased production of anti-microbial peptides which ultimately reduces acute GI-GvHD and the probability of transplant-related mortality (TRM). During inflammation, insufficient expression of VDR causes damage to the epithelial barrier and alongside, damage to paneth cells leads to reduced defensins. Compromised epithelial barrier and weak defense mechanism enhance acute GI-GvHD, and hence, increase the risk of TRM.

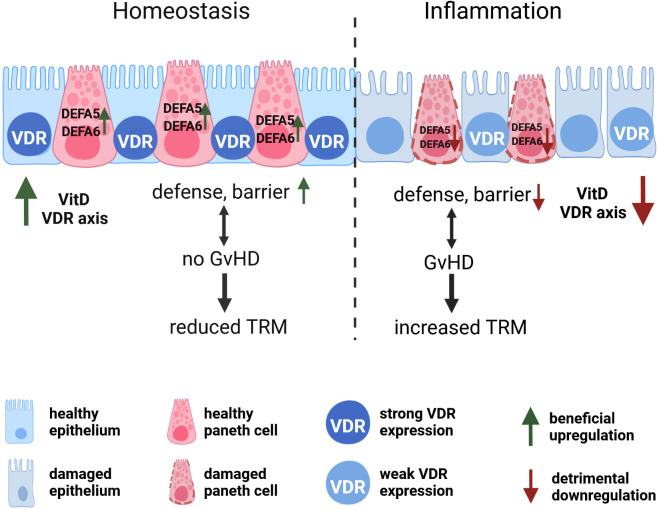

## Introduction

Although allogeneic haematopoietic stem cell transplantation (HSCT) is a potentially curative therapy for patients with hematological disorders, HSCT is still associated with substantial mortality and morbidity with up to 50% of patients at the risk of developing acute graft-versus-host disease (aGvHD) (1–3). Acute GvHD can affect skin, liver, and most severely, the upper and lower gastro-intestinal (GI) tract (2). Severe lower GI-GvHD is associated with increased treatment-related mortality (TRM), reduced survival and impaired quality of life of patients (4). Previously, we and others have linked vitamin D3 deficiency and aGvHD (5–7) and demonstrated that the 1,25-dihydroxyvitamin-D3 (1,25(OH)2D3) serum level can predict TRM and survival in HSCT patients (8). However, the reason for the protective effect of vitamin D3 is unclear.

Impaired mucosal epithelial barrier contributes significantly to the development of the inflammatory response during acute GI-GvHD. Therefore, protecting or restoring intestinal barrier function could help to mitigate acute GI-GvHD (9). Besides its well-recognized effect on skeletal health, vitamin D3 is known to be a potent immunomodulator (10–12) and maintains epithelial barrier integrity by enhancing tight junction proteins such as claudins, ZO-1, and E-cadherin (13).

The aforementioned beneficial effect of Vitamin D3 is exerted by binding of its active form to the vitamin D receptor (VDR). VDR is a nuclear hormone receptor that is a part of a steroid hormone superfamily of receptors that regulate gene transcription (14). The VDR is expressed in most cells of the immune system, including activated T lymphocytes, as well as in antigen-presenting cells (APCs) such as macrophages and dendritic cells (15). In addition, VDR is also expressed in epithelial cells (16).

The role of VDR in intestinal epithelial cells has been described by several studies with a focus on inflammatory bowel diseases (IBDs) as epidemiological data indicate an association between vitamin D3 deficiency and increased risk of IBD (17–20). Liu and colleagues demonstrated that VDR expression is reduced in patients with Crohn’s disease or ulcerative colitis and epithelial VDR signaling could inhibit colitis in a murine model independent of non-epithelial immune VDR actions (21).

In addition, Wada et al. described considerably lower expression of the VDR in the mucosa of ulcerative colitis- patients compared to normal mucosa (20). Similar results were reported by Abreu-Delgado et al. and more recently, by Garg et al. showing an inverse correlation between VDR expression and colonic inflammation in IBD patients but their studies reported conflicting data regarding the correlation between serum vitamin D3 levels and VDR expression (17, 20).

Based on the multitude of effects attributed to vitamin D3, the exact mechanism responsible for the protective effect of VDR expression in epithelial cells and/or immune cells is hard to define. Besides its role in epithelial cells and the maintenance of the intestinal mucosal barrier (13), the VDR controls the innate immune response to luminal antigens (22). Furthermore, loss of VDR signaling in lymphocytes resulted in the generation of pathogenic CD8^+^ T cells and development of IBD (23) and CD4^+^ T cells from VDR knock-out (KO) mice induced more severe colitis than wild-type CD4^+^ T cells (24). VDR has also been linked to the composition of the gut microbiota and identified as the first human gene to shape the gut microbiome (25).

Owing to the salutary effect of VDR in subsiding the inflammation and maintaining epithelial barrier integrity within the intestinal tract, we investigated the expression of *VDR* in the intestinal biopsies of HSCT patients in the absence or presence of severe acute GI-GvHD. Patients were followed up to 12 years for TRM. Herein, we report that *VDR* expression is compromised in acute GI-GvHD and associates with the outcome of transplantation.

## Materials and methods

### Patient biopsies

Gastrointestinal biopsies were obtained and analyzed from a total of 90 adult patients receiving an HSCT between October 2009 and November 2013. Patient characteristics are summarized in **Table 1**. All patients gave informed consent, the biopsy studies and scientific analyses were approved by the local ethical review board (approval no 02/220 and 09/059). All studies were performed under the regulations of Helsinki. Biopsies were either obtained in the course of a screening study in asymptomatic, clinically acute GI-GvHD-free patients (median 62 days after HSCT, n=57) or because of clinical symptoms indicative of *de novo* onset (median 78 days after HSCT, n=24) or persistence/recurrence of acute GI-GvHD (median 175 days after HSCT, n=9). Biopsies were obtained through upper or lower GI endoscopy. 33 biopsies were obtained from the small intestine and 57 biopsies from the large intestine. Clinical grading was performed according to the Glucksberg’s criteria (26) and the histological grading was performed according to the Lerner’s criteria (27).

**Table 1:** Summary of patient characteristics.

Characteristics	mean	(range)
**Age in years**		
Patients (N=90)	51	(17-71)
**Sex**	**n**	**(%)**
male	56	60
female	34	40
**Diagnosis**		
Acute leukemia	48	53
MDS/MPN	16	18
NHL	22	24
other	4	4
**Stage of underlying disease**		
early	18	20
intermediate	36	40
advanced	36	40
**Donor type**		
Unrelated donor	51	57
Sibling	38	42
Haploidentical donor	1	1
**Stem cell source**		
PBSC	81	90
BM	8	9
dCB	1	1
**Conditioning regimen**		
Reduced intensity	77	86
Full intensity	13	14
**Clinical GI-GvHD grade at the time of biopsy**		
no GvHD	46	51
Grade I	27	30
Grade II	11	12
Grade III	5	6
Grade IV	1	1
**Lerner GI-GvHD grade at the time of biopsy**		
no GvHD	43	48
Grade 1	30	33
Grade 2	12	13
Grade 3	4	5
Grade 4	1	1

MDS, myelodysplastic syndrome; MPN, myeloproliferative neoplasm; NHL, non-Hodgkin’s lymphoma; PBSC, peripheral blood stem cells; BM, bone marrow; dCB, double cord blood. Other diagnosis included aplastic anemia and Hodgkin’s lymphoma.

### RNA extraction

Total RNA was extracted from intestinal biopsies using the RNeasy Mini Kit (QIAGEN) according to the manufacturer’s recommendation. In brief, tissues were centrifuged at 4000 rpm for five minutes and were transferred to 700 µl RLT buffer (Qiagen) supplemented with 1% β-mercaptoethanol. Tissues were then sonicated for five seconds, 3-5 times, till the complete homogenization was ensured. The homogenates were filtered through QIAshredder column by centrifugation at room temperature (RT) for 3 min at 13000xg before proceeding to RNA extraction. To remove potential DNA contaminations, on-column DNA digestion with the RNase-free DNase Set (Qiagen) was implemented according to the protocol. RNA concentration was measured with ND-1000 NanoDrop Spectrophotometer (Thermo Fisher Scientific). RNA integrity quality was controlled using Agilent Bioanalyzer (Böblingen) as per the manufacturer’s instructions. RNA was stored at -80 °C until further use.

### Reverse transcription PCR (RT-qPCR)

Total RNA was reverse transcribed into complementary DNA (cDNA) using Moloney murine leukemia virus reverse transcriptase (M-MLV RT) (Promega) enzyme. Random decamers (Promega) were used to prime cDNA synthesis. The volume of 200 ng (or 1 µg when stated) of total RNA was adjusted to 13 μl with nuclease-free ddH_2_O and mixed with 1 μl Random Decamers (Promega) and 1 μl dNTPs (10 mM) on ice. Secondary structures of RNA were dissolved by 5 min incubation in thermocycler at 65°C followed by immediate incubation on ice for 1 min. After mixing with 4 μl M-MLV Buffer (5x;Promega) the samples were incubated at 42°C for 2 min. Reverse transcription started upon addition of 1 μl RT enzyme (50 min, 42°C) and was stopped by heat inactivation of the enzyme (15 min, 70°C). cDNA samples were stored at -20°C until further use.

### Gene expression using biomark fluidigm dynamic arrays

The expression of *VDR* was determined by the 48.48 Fluidigm Dynamic Arrary Integrated Fluidic Circuits (IFC) together with reference genes GAPDH and HPRT as explained elsewhere (28). The Dynamic Array™ chip was primed by injecting a control line fluid into each accumulator on opposite sides of the chip. Following this, 5 μl of each assay was loaded into the respective inlets on the left side of the chip and 5 μl of each sample was loaded into the respective inlets on the right side of the chip. The samples and assays were mixed onto the chip, in the center of the array by the IFC Controller. The chip was then run using the BioMark Gene Expression Data Collection software, according to the given parameters for the 48.48 dynamic arrays.

qPCR and melting curve analysis were performed by running the following temperature program: 2400 s at 70°C and 30 s at 60°C, followed by a hot start for 60 s at 95°C, 40 PCR cycles of 5 s at 96°C for denaturation and 20 s at 60°C for annealing and elongation. The melting curve analysis consisted of 3 s at 60°C followed by heating up to 95°C with a ramp rate of 1°C/3 s. All samples were normalized to the GAPDH, and their relative expression was calculated. Following are the primer sequences: *VDR*, Sense: GGCTACCACTTTTACATGGTCA, Antisense: TCATAATCCCGGCCTCTCTCT; *GAPDH*, Sense: TTCGACAGTCAGCCGCATC, Antisense: GCCCAATACGACCAAATCCGT. Anti-microbial peptides (AMPs) were cycled (including primer sequences) and analyzed as previously described (29).

### Immunohistochemistry

Intestinal tissue of patients with the diagnosis of no/mild acute GI-GvHD (Lerner Score 0-1) and severe acute GI-GvHD (Lerner Score 2-4) were used for this study. An *in-situ* immunohistochemical characterization and quantification (analogue to IRS-Score) of VDR were performed. For the *in-situ* characterization, standard routine diagnostic procedures and antibodies (VDR: Clone D2K6W, Cell Signaling) were used. All immunohistochemical stains were performed on tissue sections, prepared from-formalin-fixed paraffin-embedded tissue blocks. Tissues were sectioned into 2–3 µm slices. All the tissues were fixed in 4% neutral buffered formalin. Immunohistochemical staining was conducted using a Roche Ventana Benchmark Ultra automated slide stainer (Ventana Medical Systems, Roche, France). Immunofluorescence was performed as described previously (30). Briefly, after antigen retrieval, biopsies were incubated with rabbit monoclonal VDR antibody (dilution 1:100) for one hour. After washing three times with PBS, biopsies were again incubated with anti-rabbit Alexa Fluor (AF) 594 (dilution 1:100) for one hour in dark. Nuclei were counterstained with DAPI, samples were sealed with mounting media. VDR signals were observed using a Zeiss epifluorescence microscope.

### Quantification of VDR immunohistochemistry signal

Biopsies were quantified in a semi-quantitative fashion. The mucosa vitality was scored based on the presence of intact epithelium. The percentage of VDR expressing cells was scored with respect to available mucosal membrane in the biopsies. The staining score was calculated as a product of multiplication between positive cells proportion score (0-4) and the staining intensity score (0-3).

### Vitamin D3 supplementation

Until May 2012, patients received low dose Vitamin D3, i.e. 1000 to 5000 IU/d Colecalciferol. From May 2012 onwards, the patients received high dose 20.000 IU/ml Colecalciferol upon admission to the hospital followed by daily administration of 10.000 IU/ml Vitamin D3.

### Statistical analysis

Data analysis was performed in SPSS v26 (IBM Corp., Armonk, NY, USA). Test of normality was performed using the Shapiro-Wilk test. Since data were observed to be non-normally distributed, Mann-Whitney or Kruskal Wallis tests were performed and Spearman correlation coefficients were computed. Box plots were used to visualize continuous data and dot plots for semi-quantitative data. For multivariable analysis, patients were classified into two groups based on receiver-operator curve (ROC) and Youden index for best sensitivity and specificity. Area-under-the curve (AUC) was reported. Time to event was defined from day of biopsy harvest to the day of death or the last day of the patient being confirmed to be alive (censored cases). The last follow up of each patient was as recent as of January 2022. Kaplan-Meier curve was generated and log-rank test was used analyze the association of *VDR* with the risk of TRM. To further analyze the association of *VDR* expression groups with TRM, Cox regression models were applied. The multivariable Cox model was adjusted for additional covariates GI-acute GI-GvHD, age, steroids, stage of disease, and donor type. Hazard Ratios (HR) and corresponding 95%-confidence intervals (95% CI) are reported as effect estimates. To separate transplant-related hazard from relapse-related hazard, competing risk analyses were evaluated using the CumIncidence function (31).

## Results

### *VDR* gene expression downregulates in acute GI-GvHD

**Figure 1:**
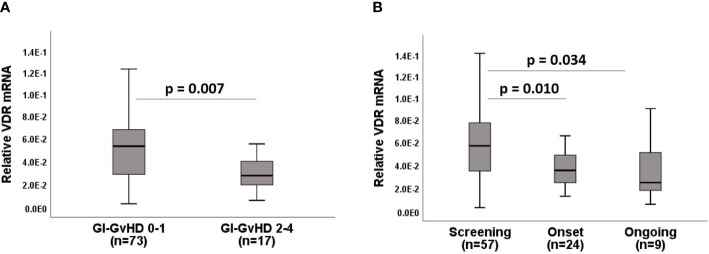
Expression of VDR mRNA in acute GI-GvHD. **(A)** VDR mRNA expression with respect to the histological classification of acute GI-GvHD. **(B)** Expression of VDR mRNA with respect to the clinical classification of acute GI-GvHD. Box plots represent median, upper and lower quartile and whiskers indicate minimal and maximal values. p value, Mann-Whitney U test.

First, we were interested in analyzing the expression of *VDR* mRNA in terms of histological acute GI-GvHD. Patients were classified into no/mild Lerner grade 0-1 and high Lerner grade 2-4 GI-GvHD. Interestingly, *VDR* mRNA expression was significantly downregulated in higher grade Lerner acute GI-GvHD patients when compared to lower grade Lerner acute GI-GvHD patients (p = 0.007, **Figure 1A**). Additionally, we also observed that patients who were supplemented with low dose vitamin D3 (1000-5000 IU/day) in our center had significantly lower intestinal *VDR* gene expression when compared to patients who were supplemented with high dose vitamin D3 (10000-20000 IU/day) within first 100 days (data not shown). As it is well-known that epithelial cells express the *VDR*, it was necessary to clarify if the *VDR* loss was solely due to epithelial denudation or if *VDR* loss could also be seen in absence of severe epithelial damage. For this purpose, we classified patients according to the clinical status of acute GI-GvHD. Clinically, GI-GvHD free patients were classified as the “screening” group, patients with a start of aGvHD were classified as the “onset” group, and the patients with progressive aGvHD were classified as the “ongoing” group. We observed that onset patients and ongoing patients displayed significantly lower *VDR* expression as compared to screening patients (p = 0.010 for onset vs. screening, p = 0.034 for ongoing vs. screening (not significant after multiple testing by Bonferroni/Sidak correction, p = 0.08), **Figure 1B**). It is important to note that aGvHD onset patients started to show GvHD did not however show severe epithelial damage. The reduction of *VDR* mRNA in onset patient samples therefore implied that *VDR* loss was evident in GvHD even when the epithelium was intact. In conclusion, *VDR* mRNA expression is significantly downregulated in histological and clinical GvHD.

### VDR protein expression is downregulated in epithelial cells during acute GI-GvHD

**Figure 2:**
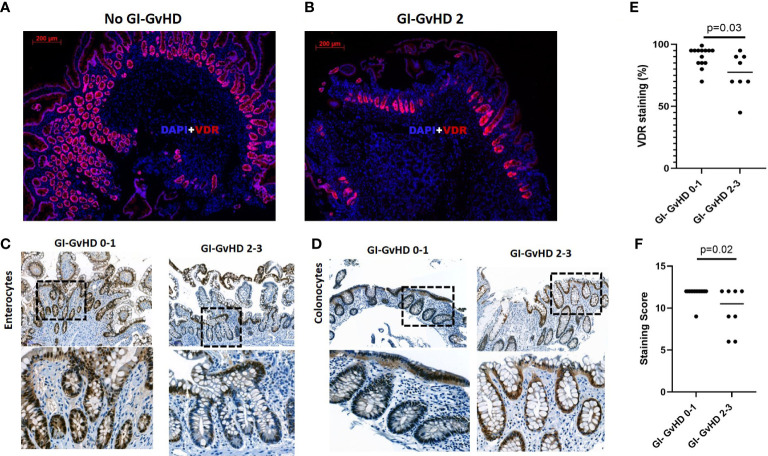
Immunofluorescence and immunohistochemistry of VDR protein in the gastro-intestinal tract of patients after allogeneic stem cell transplantation. **(A)** VDR protein expression (red) in the absence of acute GI-GvHD. **(B)** VDR protein expression in high Lerner (Grade 2) GI-GvHD. Nuclei were counterstained with DAPI (blue). Magnification: 5X objective lens. Scale bar: 200 µm. **(C)** VDR+ enterocytes in the small intestine of low and high Lerner GvHD patients. **(D)** VDR+ colonocytes in the large intestine of low and high Lerner GvHD patients in the areas with vital mucosa. Lower panel in C & D represents magnified image of upper panel from selected area. Upper panel, C&D, scale bar, 100 µm. **(E)** Semi-quantitative scoring of VDR+ cells in the gut biopsies. **(F)** Semi-quantitative scoring of VDR intensity score in the gut biopsies. Dot plots are used to represent semi quantitative score. Line represents median of the values. p value, Mann-Whitney U test.

To evaluate the protein expression of VDR, we performed immunofluorescent staining of VDR protein expression in the gut biopsies of patients and found that higher grade Lerner patients (grade 2-3) had reduced VDR expression as compared to lower Lerner patients (grade 0-1) (**Figure 2A**). Results were confirmed by single antibody immunohistochemistry (IHC) in gut biopsies of patients (lower grade Lerner 0-1, n = 15, higher grade Lerner 2-4, n=18) after HSCT. To ensure that protein differences were not influenced by GvHD- induced intestinal damage, biopsies were scored for mucosa vitality based on intact epithelium. Mucosa vitality was severely compromised in higher grade Lerner GvHD patients compared to lower grade Lerner GvHD patients (low Lerner: mean = 96%, median = 100%, min/max = 50% - 100%; high Lerner: mean = 61%, median = 60%, min/max = 10% – 95%, p= 5x10^-6^). Therefore, we made a cut-off of at least 85% mucosa vitality to ensure intact epithelium (lower grade Lerner, n=14; higher grade Lerner, n=8). An exemplary image of VDR staining from small intestine and large intestine is shown in **Figures 2C, D**. The number and intensity of VDR^+^ enterocytes in small intestine and VDR^+^ colonocytes in large intestine were strongly reduced in higher grade Lerner GvHD patients despite an intact epithelium. Of interest, we found that an overall epithelial VDR expression was significantly reduced in higher grade Lerner GvHD patients compared to lower grade Lerner GvHD patients (p = 0.03, **Figure 2E**). Furthermore, the reactivity score that evaluates the staining intensity within VDR^+^ cells was significantly reduced in higher grade Lerner GvHD (p = 0.02, **Figure 2F**). In summary, we observed that low epithelial VDR expression associated with acute GI-GvHD.

### Low *VDR* expression associates with increased transplant related mortality

**Figure 3:**
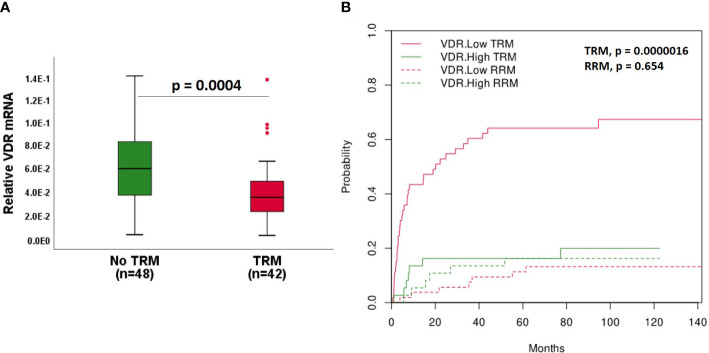
VDR expression with respect to TRM. **(A)** VDR gene expression in patients without and with TRM. Median survival time in: No TRM = 88 months, TRM = 11 months. **(B)** Association of VDR with cumulative incidence function estimates for competing risk data. Patients were classified according to high (green line) and low (red line) expression of VDR based on ROC curve. The cumulative risk of TRM and RRM with respect to months after biopsy retrieval is shown. Box plots represent the median, upper and lower quartile and whiskers indicate minimal and maximal values. p value, Mann-Whitney U test.

To examine the impact of *VDR* expression on prognosis after HSCT, we categorized patients into the groups who died as a consequence of TRM and who did not display TRM. Of note, patients dying of infection, acute GI-GvHD or toxicity were compounded as TRM group. We found a significant downregulation of *VDR* mRNA expression in the patient group who exhibited TRM compared to patients without TRM (median survival time in no TRM group= 88 months, median survival time in TRM group = 11 months, p = 4x10^-4^, **Figure 3A**). In order to deal with varying length of observation times, a defined 1-year TRM from the biopsy retrieval was additionally analyzed. Again, we observed a strong reduction of *VDR* mRNA in the TRM group when compared to the non-TRM group (median survival time in no TRM group = 12 months, median survival time in TRM group = 3 months, p = 0.018, **Supplementary Figure 1A**). To further address this association, we classified patients based on high and low *VDR* expression. The point within the ROC curve with highest sensitivity and specificity was chosen for dichotomization of *VDR* mRNA expression. The AUC was 0.714 (95% CI = 0.604 – 0.824, p = 4x10^-4^). The Kaplan-Meier survival curve revealed a significantly higher probability of TRM in patients with low *VDR* expression (median survival time in *VDR* low group = 14 months, median survival time in *VDR* high group = 67 months, log-rank p = 1x10^-5^, **Supplementary Figure 1B**). In the context of HSCT, the relapse related mortality (RRM) is a competing event for TRM. In our patient cohort, 13 patients died of relapse. Therefore, we performed competing risk analyses to account for RRM as a competing event. We observed a significant association of low *VDR* expression with the probability of TRM (p = 1.6x10^-6^, **Figure 3B**). In contrast, no significant association of *VDR* expression with relapse was observed (p = 0.654, **Figure 3B**).

To determine the potency of *VDR* as an independent risk factor for TRM, a Cox regression model was performed. In the unadjusted model, low *VDR* expression and higher grade Lerner acute GI-GvHD (grade 2-4) were associated with the risk of TRM (**Table 2**). After adjustment for classical risk factors, multivariate Cox regression showed low *VDR* expression (p = 0.001, hazard ratio 4.14, 95% CI 1.78 – 9.63) and higher grade Lerner acute GI-GvHD (grade 2-4) (p = 0.001, hazard ratio 2.65, 95% CI 1.33 – 5.31) as an independent risk factor for TRM (**Table 2**). Classical risk factors such as age, disease status, donor type were not found to be significantly associated. When we looked at 1-year outcome from the time of biopsy, we again observed low *VDR* and severe acute GI-GvHD as a risk factor for TRM (**Supplementary Table 1**). Although steroid treatment seemed to suppress *VDR* expression in univariate and multivariate analyses (data not shown), Cox regression analyses demonstrated that steroid treatment is not a risk factor for TRM. Taken together, the data indicates that low *VDR* expression predicts outcome after HSCT.

**Table 2:** Results from Cox regression analysis for TRM.

	HR	95% CI for HR	P
**Unadjusted (univariable)**			
**Low VDR** **(n = 53)**	4.90	2.16 – 11.08	**0.00013**
**Grade 2-4 acute GI-GvHD** (n = 17)	3.09	1.61 – 5.93	**0.001**
**Adjusted (multivariable)**			
**Low VDR** (n = 53)	4.14	1.78 – 9.63	**0.001**
**Grade 2-4 acute GI-GvHD** (n = 17)	2.65	1.33 – 5.31	**0.006**
**Steroid** (patients on steroids, n = 51)	1.62	0.82 – 3.20	0.16
**Stage of disease** (advanced, n = 36)	1.78	0.87 – 3.64	0.11
**Patient´s age** (> 50 years, n = 58)	1.09	0.54 – 2.21	0.80
**Donor type** (MUD, n = 51)	1.05	0.52 – 2.12	0.88

Out of the 90 patients, 42 died of TRM, 13 died of RRM and 35 lived until end of observation period (maximum follow-up time = 142 months, median follow-up time = 34 months).

High-risk group with respective patients numbers are given for categorical variables. HR, hazard ratio; CI, confidence interval; MUD, matched unrelated donor. Significant differences in p values are shown in bold numbers.

### *VDR* expression correlates with anti-microbial peptides

**Figure 4:**
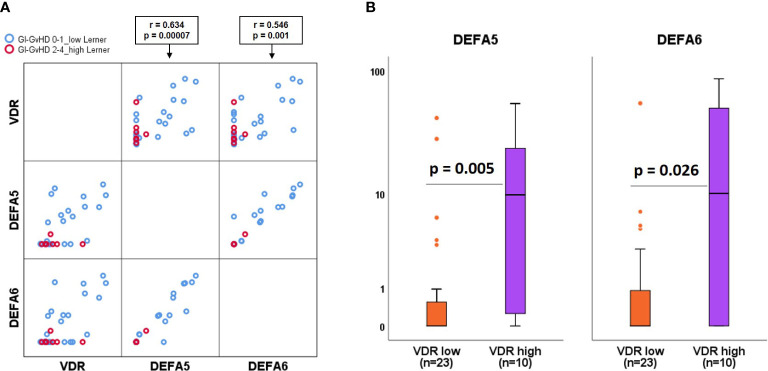
Association of anti-microbial peptides with VDR. **(A)** Matrix scatter correlation plot of VDR, DEFA5, DEFA6 genes within low (blue circles) and high (red circles) Lerner GvHD. **(B)** Comparison of alpha defensins gene expression in patients with low versus high VDR gene expression. Box plots represent median, upper and lower quartile and whiskers indicate minimal and maximal values. r value, Spearmann correlation coefficient. p value, Mann-Whitney U test.

Anti-microbial peptides (AMPs) are known downstream targets of the VitD3-VDR pathway. Paneth cells express AMPs and are known to be expressed abundantly in the small intestine (29). Therefore, we investigated the correlation between *VDR* and AMPs gene expression in small intestinal biopsies in our patient cohort. We found a significant correlation of *VDR* expression with defensins: *DEFA5*, *DEFA6* genes (**Figure 4A**). Within the correlation plot, it can be visualized that lower grade Lerner patients show high *VDR* and AMPs (blue circles) and higher grade Lerner patients show low *VDR* and AMPs (red circles) gene expression. To further substantiate the relation between *VDR* and AMPs, we tested the AMPs expression comparing patients with low and high *VDR* expression. As expected, we found low AMPS in “*VDR* low” patients while “*VDR* high” patients showed significantly high AMPs (*DEFA5*: p = 0.005, *DEFA6*: p = 0.026, **Figure 4B**).

## Discussion

The central aim of this study was to investigate the possible role of VDR in the context of HSCT, with a focus on acute GI-GvHD. We found a significant reduction of *VDR* gene expression in severe acute GI-GvHD patients compared to no/mild acute GI-GvHD, suggesting an important role of VDR in the context of HSCT. Moreover, we demonstrated that patients with lower expression of *VDR* mRNA had significantly increased risk of TRM independent classical risk factors. Our results suggest that *VDR* gene expression associates with TRM and therefore may help to predict the outcome of allogeneic stem cell transplantation.

Our findings are novel in the field of acute GI-GvHD. Other clinical studies, outside the GvHD context, have reported the loss of intestinal VDR in intestinal inflammation such as ulcerative colitis and Crohn’s disease subjects (20, 21) which are in line with our observation. Previous findings reported that VDR protein expression in the colonic mucosa of ulcerative colitis patients was significantly lower than the normal colon mucosa (20). Similarly, the loss of VDR protein and the downregulation of *VDR* transcript in the colonic biopsies of IBD patients was previously observed in an inter-continental cohort (21). More recently, a decreased level of *VDR* mRNA in the colonic epithelial cell lines under an inflammatory situations was reported (32). Within our patient cohort, the downregulation of *VDR* expression in GvHD patients’ intestinal biopsies points toward the importance of the Vitamin D3-VDR pathway which is abrogated in GvHD-related inflammation.

Since epithelial cells express *VDR*, one can argue that the loss of *VDR* in acute GI-GvHD is in fact due to the loss of epithelium. However, when we compared clinical acute GI-GvHD free screening patients with the clinical onset patients without substantial loss of epithelium, we still found a significant reduction of *VDR* mRNA in onset patients. This finding implicates that *VDR* loss is independent of epithelial denudation. This finding was further supported by immunohistochemistry of VDR in lower grade Lerner and higher grade Lerner GvHD patients without severe epithelial damage. We observed significant downregulation of VDR signals in higher grade Lerner patients compared to lower grade Lerner patients. This observation implicated an inability of the gut epithelium to express sufficient VDR under inflammatory conditions such as GvHD. Although previous research highlighted the role of micro RNA 346 and zinc finger protein 36 in the suppression of VDR expression in epithelial cells (32, 33), the precise molecular mechanism behind such phenomenon in GvHD patients’ gut is yet to be clarified.

We observed a strong association of *VDR* with the outcome after HSCT. One main reason for poor outcome in aGvHD is the damaged epithelial barrier. The role of VDR in epithelial barrier maintenance has been previously described in several non-GvHD inflammatory diseases (13, 21). The mechanism behind this phenomenon involves the induction of tight junction proteins in epithelial cells and the suppression of NF- κB pathway, leading to a reduction of epithelial apoptosis. Another possible mechanism involves the VDR mediated protection of intestinal stem cells (ISC). It is known that the VDR is expressed by LGR5^+^ stem cell in patients’ intestine (34). In a murine model, VDR deficiency impaired intestinal stem cell proliferation and suppressed epithelial regeneration (35). In line, Vitamin D3 rich diet led to higher count of Lgr5^+^ stem cells (36). Therefore, we propose that the beneficial effect of VDR could be partially mediated *via* ISC protection. The higher probability of TRM in patients with a low level of *VDR* is further supported by the multivariable Cox regression model where low *VDR* was an independent risk factor to predict TRM, which, interestingly, was independent of the presence of severe GvHD. Steroid use did not predict TRM but did have an effect on suppression of *VDR* expression in univariate and multivariate analyses and seems to have an independent effect that cannot be explained by GvHD. The results suggest that low *VDR* predicts the probability of TRM without competing with relapse events. These data suggest that VDR might represent a potential tissue biomarker in GvHD, and, high VDR expression could probably confer protection in GvHD. This makes VDR an attractive tool for GvHD therapy.

Recently, Lu et al. showed that VDR is necessary for the optimal function of Paneth cells (37). In the present study, we found a strong correlation and association of alpha-defensins with *VDR* expression that highlights the importance of the Vitamin D3-VDR axis in Paneth cell function. It can be hypothesized that the VDR signaling is crucial for the maintenance of Paneth cells in GvHD patients. Moreover, the expression of VDR expression in enterocytes and colonocytes within the GI tract points toward the strong necessity of the VDR pathway to maintain gut epithelial integrity which is in line with previous findings (13, 38).

Following our previous finding that the active Vitamin D3 metabolite in the serum on day -2 to day 7 peritransplant could help predict 1-year TRM and 1-year survival (8), we opted to correlate vitamin D3 serum level and the intestinal *VDR* expression. We had no access to gut biopsies within day -2 to day 7 which is the period when patients are most vulnerable to infections due to immunosuppressant and prophylactic regimens. Also, active vitamin D3 (calcitriol) has a half-life of 15 hours (39), which indicates that the biopsy retrieval and serum vitamin D3 measurement should go parallel. In addition, it is not clear whether circulating serum vitamin D3 levels reflect the local abundance of vitamin D3 metabolites in the tissues. Nevertheless, a direct comparison of serum vitamin D3 and intestinal VDR could shed more light on the importance of VitD3-VDR pathway and expand our understanding of the mechanistic insights of VDR in transplanted patients.

Our data clearly show that patients with sufficient *VDR* expression have a higher probability to be protected from transplant-associated complications which could partly be interpreted as an ability of epithelial tissue to be resilient, regenerative, and able to tolerate an insult mediated by allogeneic T cells. The role of VDR in tissue tolerance is an open discussion and, in fact, a need for future research. In addition, to further validate *VDR* expression as a marker for acute GI-GvHD development, different cohorts should be investigated.

In summary, in this study, we present clinical evidence suggesting that the VDR plays a critical and multifaceted role in acute GI-GvHD and mortality. Patients lacking an optimal expression of VDR in epithelial cells of the gut are susceptible to mucosal damage following acute GI-GvHD aggravation and ultimately poor outcomes. High-dose Vitamin D3 supplementation might be one of the rescue mechanisms to induce VDR expression. Therefore, the supplementation of patients undergoing HSCT may be beneficial. Other strategies and interventions concerning VDR induction are urgently required in the context of GvHD and other conditions where gut inflammation is involved. In conclusion, targeting VDR may provide patients with disease amelioration and a better quality of life.

## Data availability statement

The original contributions presented in the study are included in the article/Supplementary Materials. Further inquiries can be directed to the corresponding author.

## Ethics statement

The studies involving human participants were reviewed and approved by Aktive Ethikvoten der Ethik-Kommissionan der Universität Regensburg. Email: ethikkomission@ur.de. The patients/participants provided their written informed consent to participate in this study.

## Author contributions

CM analyzed the data, wrote and reviewed the manuscript, AM performed immunohistochemistry and reviewed the manuscript, PS designed the study, performed qPCR experiments and reviewed the manuscript, EM and DWe provided clinical data and reviewed the manuscript, AH performed competing risk analysis and reviewed the manuscript, SarG, AG, AD, RD, LW, HP, ME, DWo, and WH discussed the data and reviewed the manuscript, KS and IH supported the statistics and reviewed the manuscript, EH and MK designed the study, discussed the data and reviewed the manuscript, SakG designed the study, supervised the project, performed experiments, analyzed data, and wrote the manuscript. All authors contributed to the article and approved the submitted version.

## Funding

This work was supported by European Union Grant FP7-PEOPLE-2012-ITN-315963 (CELLEUROPE), Wilhelm Sander Foundation, Grant 2017.020.1 “Dysbiosis and intestinal immunoregulation in GvHD”, the Deutsche Forschungsgemeinschaft (DFG, German Research Foundation) - Projektnummer 324392634 - TRR 221, CRC 1371 microbiome signatures, German Jose Carerras Leukemia Foundation (grant DJCLS 01 GvHD/2016), Else-Kröner-Fresenius Stiftung and the European Cooperation in Science & Technology under the COST Action CA17138 (Integrated European Network on Chronic Graft Versus Host Disease: EUROGRAFT).

## Acknowledgments

We acknowledge our technicians Doris Gaag, Heike Bremm, Massimiliano Caioni, Tatjana Schifferstein and Yvonne Schumann for the excellent technical support.

## Conflict of interest

The authors declare that the research was conducted in the absence of any commercial or financial relationships that could be construed as a potential conflict of interest.

## Publisher’s note

All claims expressed in this article are solely those of the authors and do not necessarily represent those of their affiliated organizations, or those of the publisher, the editors and the reviewers. Any product that may be evaluated in this article, or claim that may be made by its manufacturer, is not guaranteed or endorsed by the publisher.

## Supplementary material

Supplementary Table 1 Results from Cox regression analysis for 1-year TRM. Out of the 90 patients, 28 died of TRM, 4 died of RRM and 58 lived until end of 1-year observation period. High-risk group with respective patients numbers are given for categorical variables. HR, hazard ratio; CI, confidence interval; MUD, matched unrelated donor. Significant differences in p values are shown in bold numbers.

Supplementary Figure 1 Association of VDR gene with the probability of TRM. A. VDR gene expression without and with TRM within 1-year following the biopsy retrieval. Median survival time in no TRM group = 12 months, median survival time in TRM group = 3 months. B. Kaplan-Meier curve showing the probability of TRM in patients with low (red line) or high (green line) VDR gene expression. The time in x-axis refers to the time from obtaining biopsies until the last follow up. Median survival time in VDR low group = 14 months, median survival time in VDR high group = 67 months. Box plots represent median, upper and lower quartile and whiskers indicate minimal and maximal values. p value, Mann-Whitney U test for A and log rank test for B.
