# Supplementary material for: Downregulation of the vitamin D receptor expression during acute gastrointestinal graft versus host disease is associated with poor outcome after allogeneic stem cell transplantation
Source: Front Immunol. 2022 Oct 20;13:1028850. doi: 10.3389/fimmu.2022.1028850 (PMC9632171; doi:10.3389/fimmu.2022.1028850)
Supplement: Supplementary Table 1 — Results from Cox regression analysis for 1-year TRM. Out of the 90 patients, 28 died of TRM, 4 died of RRM and 58 lived until end of 1-year observation period. High-risk group with respective patients numbers are given for categorical variables. HR, hazard ratio; CI, confidence interval; MUD, matched unrelated donor. Significant differences in p values are shown in bold numbers. [file Table_1.docx]

Supplementary Table 1. Results from Cox regression analysis for 1-year TRM. Out of the 90 patients, 28 died of TRM, 4 died of RRM and 58 lived until end of 1-year observation period.

|  | HR | 95% CI for HR | P |
| --- | --- | --- | --- |
| Unadjusted (univariable) | | | |
| Low VDR  (n = 53) | 4.07 | 1.54 – 10.74 | **0.004** |
| Grade 2-4 acute GI-GvHD  (n = 17) | 3.12 | 1.43 – 6.78 | **0.004** |
| Adjusted (multivariable) | | | |
| Low VDR  (n = 53) | 2.91 | 1.06 – 7.99 | **0.038** |
| Grade 2-4 acute GI-GvHD  (n = 17) | 2.69 | 1.12 – 6.46 | **0.026** |
| Steroid  (patients on steroids, n = 51) | 2.37 | 0.98 – 5.75 | 0.055 |
| Stage of disease  (advanced, n = 36) | 2.10 | 0.87 – 5.02 | 0.095 |
| Patient´s age  (> 50 years, n = 58 ) | 1.35 | 0.54 – 3.36 | 0.50 |
| Donor type  (MUD, n = 51) | 1.50 | 0.61 – 3.67 | 0.36 |
