# Supplementary figures and images for: Downregulation of the vitamin D receptor expression during acute gastrointestinal graft versus host disease is associated with poor outcome after allogeneic stem cell transplantation
Source: Front Immunol. 2022 Oct 20;13:1028850. doi: 10.3389/fimmu.2022.1028850 (PMC9632171; doi:10.3389/fimmu.2022.1028850)

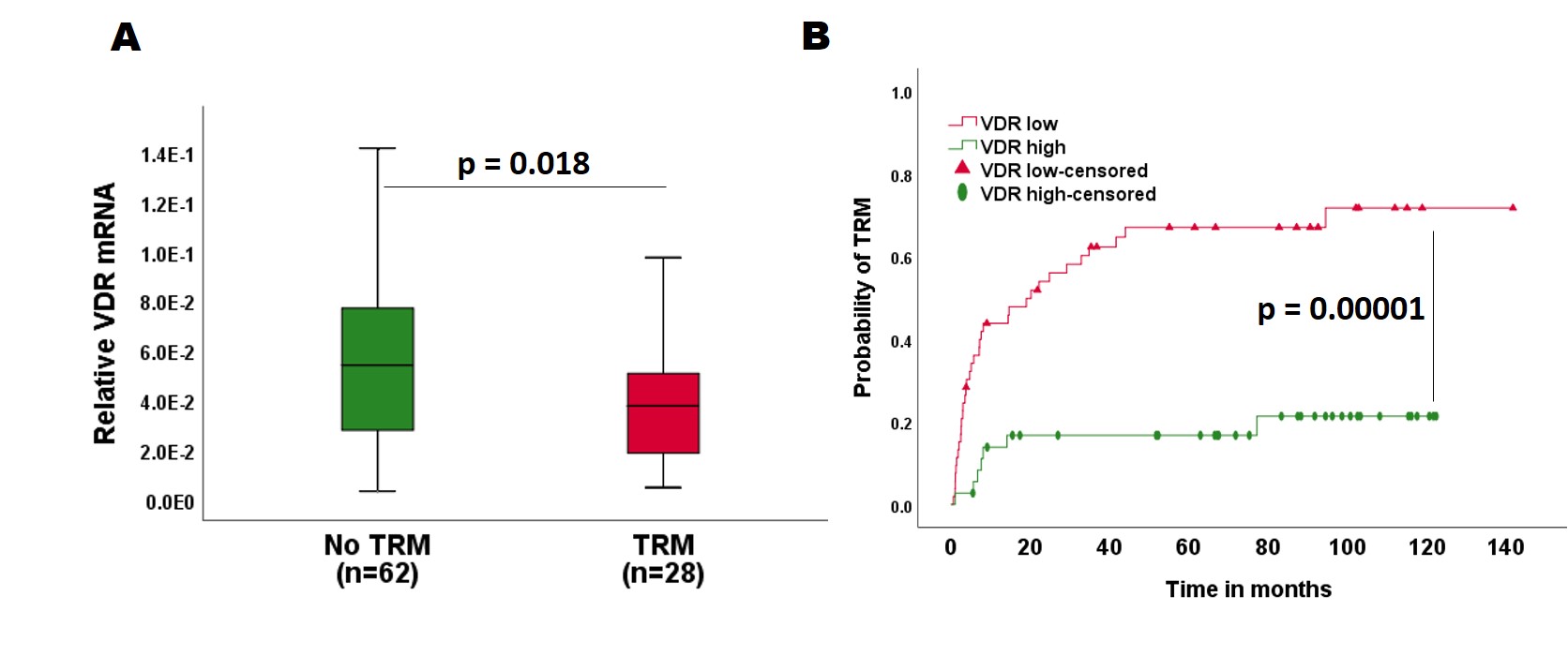

Supplement: Supplementary Figure 1 — Association of VDR gene with the probability of TRM. A. VDR gene expression without and with TRM within 1-year following the biopsy retrieval. Median survival time in no TRM group = 12 months, median survival time in TRM group = 3 months. B. Kaplan-Meier curve showing the probability of TRM in patients with low (red line) or high (green line) VDR gene expression. The time in x-axis refers to the time from obtaining biopsies until the last follow up. Median survival time in VDR low group = 14 months, median survival time in VDR high group = 67 months. Box plots represent median, upper and lower quartile and whiskers indicate minimal and maximal values. p value, Mann-Whitney U test for A and log rank test for B. [file Image_1.jpeg]
